# Persistent immune activation and altered gut integrity over time in a longitudinal study of Ugandan youth with perinatally acquired HIV

**DOI:** 10.3389/fimmu.2023.1165964

**Published:** 2023-03-28

**Authors:** Sahera Dirajlal-Fargo, Monika Strah, Kate Ailstock, Abdus Sattar, Christine Karungi, Rashidah Nazzinda, Cissy Kityo, Victor Musiime, Nicholas Funderburg, Grace A. McComsey

**Affiliations:** ^1^ Division of Pediatrics, University Hospitals Cleveland Medical Center, Cleveland, OH, United States; ^2^ Division of Infectious Diseases, Rainbow Babies and Children’s Hospital, Cleveland, OH, United States; ^3^ School of Medicine and Department of Population and Quantitative Health Sciences, Case Western Reserve University, Cleveland, OH, United States; ^4^ School of Health and Rehabilitation Sciences, Ohio State University School of Health and Rehabilitation Sciences, Columbus, OH, United States; ^5^ Joint Clinical Research Centre, Kampala, Uganda; ^6^ Makerere University, Kampala, Uganda

**Keywords:** inflammation, immune activation, gut integrity, microbial translocation, children, HIV, sub-Saharan Africa

## Abstract

**Introduction:**

Perinatally acquired HIV infection (PHIV) occurs during a critical window of immune development. We investigated changes in systemic inflammation and immune activation in adolescents with PHIV and those without HIV (HIV-) in Uganda.

**Methods:**

A prospective observational cohort study was performed in 2017-2021 in Uganda. All participants were between 10-18 years of age and without active co-infections. PHIVs were on ART with HIV-1 RNA level ≤400 copies/mL. We measured plasma and cellular markers of monocyte activation, T-cell activation (expression of CD38 and HLA-DR on CD4+ and CD8+), oxidized LDL, markers of gut integrity and fungal translocation. Groups were compared using Wilcoxon rank sum tests. Changes from baseline were examined with 97.5% confidence intervals on relative fold change. P values were adjusted for false discovery rate.

**Results:**

We enrolled 101 PHIV and 96 HIV-; among these, 89 PHIV and 79 HIV- also had measurements at 96 weeks. At baseline, median (Q1, Q3) age was 13 yrs (11,15), and 52% were females. In PHIV, median CD4+ cell counts were 988 cells/µL (638, 1308), ART duration was 10 yrs (8, 11), and 85% had viral load <50 copies/mL throughout the study, 53% of participants had a regimen switch between visits, 85% of whom switched to 3TC, TDF and DTG. Over 96 weeks, while hsCRP decreased by 40% in PHIV (p=0.12), I-FABP and BDG both increased by 19 and 38% respectively (p=0.08 and ≤0.01) and did not change in HIV- (p≥0.33). At baseline, PHIVs had higher monocyte activation (sCD14) (p=0.01) and elevated frequencies of non-classical monocytes (p<0.01) compared to HIV- which remained stable over time in PHIV but increased by 34% and 80% respectively in HIV-. At both time points, PHIVs had higher T cell activation (p ≤ 0.03: CD4+/CD8+ T cells expressing HLA-DR and CD38). Only in PHIV, at both timepoints, oxidized LDL was inversely associated with activated T cells(p<0.01). Switching to dolutegravir at week 96 was significantly associated an elevated level of sCD163 (β=0.4, 95% CI=0.14,0.57, p<0.01), without changes in other markers.

**Conclusion:**

Ugandan PHIV with viral suppression have some improvement in markers of inflammation over time, however T-cell activation remains elevated. Gut integrity and translocation worsened only in PHIV over time. A deeper understanding of the mechanisms causing immune activation in ART treated African PHIV is crucial.

## Introduction

Adolescents are a growing population of interest in HIV and our understanding of ongoing immune dysregulation in this population is lacking. Wide access to antiretroviral therapy (ART) has transformed HIV from a fatal condition into a chronic disease. Despite the high prevalence of HIV in children living in sub-Saharan Africa, there are limited data on the activation status of immune cells in perinatally acquired HIV (PHIV) in the setting of ART. Perinatal HIV transmission refers to HIV transmission from mother to child during either pregnancy, labor and delivery, or breastfeeding and accounts for the majority of childhood HIV infections. With the universal recommendation for ART treatment, understanding the side effects of ART exposure and the immunologic perturbations that persist despite viral suppression in PHIV is paramount to reducing long-term complications as children age into early adulthood. There is a need for further studies in African PHIV and for exercising caution when extrapolating data from PHIV in the global North to PHIV in the global South.

We have previously assessed the consequences of PHIV and its treatment with ART in a cohort of adolescents in Uganda (1–5) and have reported significant differences in the immune profiles of HIV- and PHIV children. In this study, we assessed changes over 96 weeks of sustained ART in soluble and cellular markers of monocyte activation and T-cell activation, plasma markers of systemic inflammation, oxidized lipids, and gut integrity in youth with PHIV, in comparison to age and sex matched Ugandan youth without HIV (HIV-). The primary objectives of this study were to 1) assess changes in inflammatory biomarkers over 96 weeks by study arm; and 2) to determine the association between gut integrity/fungal translocation, oxidized lipids and T-cell and monocyte activation over time 3) and lastly explore in this pediatric cohort, followed since 2017, immune changes pre- and post- integrase strand transfer inhibitors (INSTIs).

## Methods

### Study design

This is a prospective observational cohort of PHIV and HIV- children prospectively enrolled at the Joint Clinical Research Center (JCRC) in Kampala, Uganda between 2017 and 2021. The study was approved by the Research Ethics Committee in Uganda, the Ugandan National Council of Science and Technology as well as the IRB of the University Hospitals Cleveland Medical Center, Cleveland, Ohio. Caregivers gave written informed consent. All participants were 10-18 years of age and they provided written informed assent as per research guidelines in Uganda. Timing of HIV acquisition in PHIV participants could not be ascertained but started *in utero*, at birth or shortly thereafter. PHIV participants were on ART for at least 2 years with a stable regimen for at least the last 6 months with HIV-1 RNA < 400 copies/mL. Evidence of self-reported or documented diarrhea or acute infection (malaria, tuberculosis, helminthiasis, pneumonia, meningitis) in the last 3 months, as well as moderate or severe malnutrition were exclusionary. Adolescents with pregnancy or intent to become pregnant were excluded. All participants lived in Kampala and surrounding areas (urban or peri-urban).

### Study evaluations

Participants were seen at study entry (baseline) and at week 96 during which a Ugandan pediatrician (RN) performed a physical exam and measured body mass index (BM)I and sexual maturity rating or Tanner staging. Blood was drawn after at least an 8-hour fast. Blood was processed and plasma, serum, and PBMCs were cryopreserved for shipment to University Hospitals Cleveland Medical Center, Cleveland, Ohio. All assays were performed in batches and without prior thaw.

### Cellular markers of monocyte and T-cell activation

Monocytes and T-cells were phenotyped by flow cytometry as previously described by Dr. Funderburg (6). CD4^+^ and CD8^+^ T-cell activation was measured by expression of CD38 and HLA-DR. Monocyte subsets were determined by the relative expression of CD14 and CD16.

### Inflammation, soluble immune activation and gut markers

We selected intestinal biomarkers based on prior data in PHIV in Uganda as well as in adults living with HIV suggesting a potential role in cardiovascular disease (CVD) and inflammation (1). Beta D glucan (BDG, Mybiosource Inc. CA), is a polysaccharide cell wall component of most fungal species. Intestinal fatty acid binding protein (I-FABP, R &D Systems, Minneapolis, Minnesota, USA) is considered a marker of enterocyte inflammation or damage (7, 8). Soluble CD14 (sCD14, R &D Systems, Minneapolis, Minnesota) is a marker of monocyte activation, and is associated with mortality and progression of atherosclerosis[26]. We have shown that oxidized lipids (oxLDL) upregulates monocyte activation in HIV (9), making oxLDL a potentially important mediator on the causal pathway of monocyte activation.

sCD163, hsCRP, IL6 and ox LDL were measured by ELISA (R &D Systems, Minneapolis, Minnesota, USA, ALPCO, Salem, New Hampshire, USA and Mercodia, Uppsala, Sweden). The intra-assay variability ranged between 4-8% and inter-assay variability was less than 10% for all markers. All assays were performed at Dr. Funderburg’s laboratory at Ohio State University, Columbus, OH. Laboratory personnel were blinded to group assignments.

### Statistical analysis

All variables were compared between groups using Wilcoxon rank sum tests or Fisher’s exact tests, as appropriate. Biomarkers were analyzed baseline and at week 96. Biomarker changes over time were calculated as the mean difference between week 96 values and baseline values on the log10 scale and back transformed to represent mean fold-change from baseline. Evidence for change over time are presented with 97.5% confidence intervals (CI), where 1 indicates no change. Shifts in the distribution of changes from baseline were evaluated using Wilcoxon rank sum tests and described as relative fold-change with corresponding p-values. FDR adjusted p-values using the Benjamini-Hochberg method to control for false discovery rates are also provided. The relationship between inflammatory markers and predictor variables of interest were assessed using Spearman correlation analyses. We fitted generalized estimating equations (GEE) with unstructured correlations on the inflammatory biomarkers over 96 weeks to estimate the effect of medication switch and dolutegravir. The GEE models included terms for observation time, medication switch, and an interaction term medication switch x time. Models were fit for each biomarker separately. We then estimated the effect of switching to dolutegravir with GEE models by including terms for observation time, switch to dolutegravir and an interaction terms dolutegravir switch x time. All statistical analyses were performed using R 4.2.1 and STATA 17.0 BE

## Results

### Participant characteristics

Baseline characteristics are highlighted in **Table 1**. Of the 197 participants recruited at baseline (101 PHIV, 96 HIV-), 168 (89 PHIV, 79 HIV-) had measurements at 96 weeks. Reasons for drop out before 96 weeks included loss to follow-up (n=10), one pregnancy, fear/inability to come to the clinic during the first wave of the COVID-19 pandemic (n=10) and relocation (n=9). Median age at enrollment was 13 years and 53% of participants were female. All participants, regardless of HIV status, were exposed to high levels of socioeconomic adversity. PHIV were more likely to have lack of access to clean water and electricity. Over the study period, 30% of participants went through puberty and reached Tanner stage 4-5.

**Table 1:** Baseline characteristics.

	PHIV N=101	HIV-N=96	*p*
Age (years)	12.93 [11.53, 14.71]	12.67 [11.08, 14.33]	0.24
Female sex (%)	54 (53)	50 (52)	0.96
BMI-for-age z score	-0.57 [-1.27, -0.02]	-0.34 [-1.05, 0.33]	0.10
Socioeconomic factors			
Living in extreme poverty (<$1.90/day, %)	42 (56)	37 (49%)	0.46
Food Scarcity (%)	26 (25)	37 (37)	0.10
No electricity (%)	22 (22)	11 (12)	0.08
Unprotected water source (%)	14 (14)	2 (2)	<0.01
HIV variables			
Viral load< 20 copies/mL (%)	84 (86)		
CD4 nadir (cells/µL)	619.50 [333, 1097]		
CD4 cell count (cells/µL)	988 [638, 1307]		
CD4 percent	34.50 [27, 41]		
ART Duration (years)	9.88 [7.61, 11.08]		
Nucleotide Reverse Transcriptase Inhibitor (%)			
Abacavir	42 (47)		
Lamivudine	1 (1)		
Tenofovir	12 (14)		
Zidovudine	33 (37)		
Nevirapine (%)	18 (27)		
Efavirenz (%)	46 (44)		
Lopinavir/ritonavir (%)	27 (28)		
Soluble Markers of Systemic Inflammation and oxidative stress			
hsCRP (ng/mL)	515.92 [185.69, 1619.31]	398.60 [126.77, 1145.82]	0.16
IL6 (pg/mL)	1.13 [0.77, 2.12]	1.23 [0.84, 1.86]	0.93
OxLDL	40089.82 [34254.01, 47496.50]	44827.71 [37387.94, 52520.84]	**0.016**
Gut integrity and translocation markers			
IFAB-P (pg/mL)	2151.44 [1701.91, 3245.97]	2241.48 [1687.72, 2966.32]	0.49
BDG (pg/mL)	283.66 [230.07, 488.86]	448.65 [294.19, 613.45]	**<0.001**
Markers of Monocytes and T-Cell activation			
sCD14 (pg/mL)	2110.70 [1759.38, 2568.92]	1675.39 [1445.09, 1956.71]	**<0.01**
sCD163 (pg/mL)	589.60 [404.24, 735.95]	684.97 [495.51, 854.47]	**0.01**
%MNC: CD14+CD16-	.68 (.59-.78)	.71 (.64-.77)	0.78
%MNC: CD14^dim^CD16^+^	0.08 [0.06, 0.11]	0.07 [0.04, 0.09]	**0.023**
%MNC: CD14^+^CD16^+^	0.22 [0.15, 0.30]	0.23 [0.17, 0.27]	0.958
%CD4^+:^ CD38^+^	11.57 [10.18, 13.19]	11.66 [9.86, 13.21]	0.978
%CD4+: HLA-DR^+^	11.25 [9.59, 12.67]	9.24 [7.58, 10.15]	**<0.01**
%CD4^+:^ CD38^+^HLA-DR^+^	4.10 [2.89, 5.28]	3.58 [2.60, 4.64]	0.088
%CD8+: CD38^+^	8.06 [6.77, 10.25]	7.66 [6.04, 9.45]	0.342
%CD8^+:^ HLA-DR^+^	8.11 [7.25, 9.43]	7.16 [6.25, 7.82]	**<0.01**
%CD8^+:^ CD38^+^HLA-DR^+^	8.93 [6.71, 14.69]	8.29 [5.79, 12.69]	0.23

Median [Interquartile Range], Bold values represent p<0.05, ART, antiretroviral therapy; PHIV, children with perinatally acquired HIV; MNC, monocytes.Bold values represent p values <0.05.

At baseline, 86% PHIV had viral load < 50 copies/mL and remained undetectable at week 96. At baseline 72% were on a non-nucleotide reverse transcriptase inhibitor (NNRTI) containing regimen, 28% were on lopinavir/ritonavir (LPV/r) and 2 participants were on dolutegravir; 71% had a past history of thymidine analogue exposure (AZT or D4T). During the 96 week study period, 53% of participants had drug substitutions, 85% of whom switched to lamivudine, tenofovir and dolutegravir (3TC, TDF and DTG) for regimen optimization based on the Ugandan Ministry of Health HIV Guidelines (10).

Median ART duration at baseline was 10 years, with 8 participants initiating ART under the age of 1 and the rest between the ages of 2 and 10. All but 3 PHIV were on cotrimoxazole prophylaxis throughout the study and none of the participants were receiving antifungal or tuberculosis medications.

### Changes in markers of systemic inflammation, gut integrity and oxidative lipids

**Figure 1:**
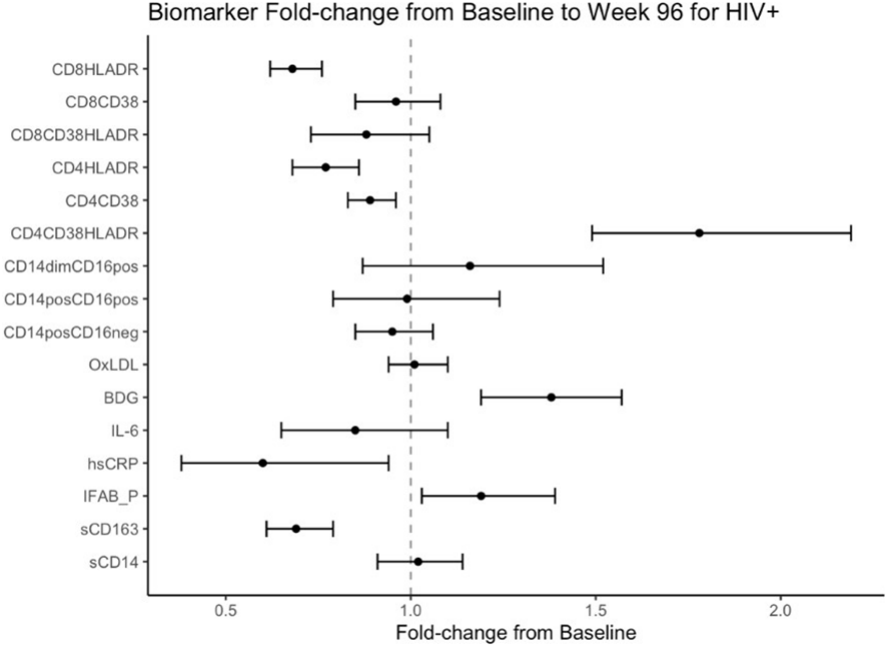
Fold Change from baseline over 96 weeks. Point estimates and error bars reflect mean and 95% confidence intervals.

**Figure 2:**
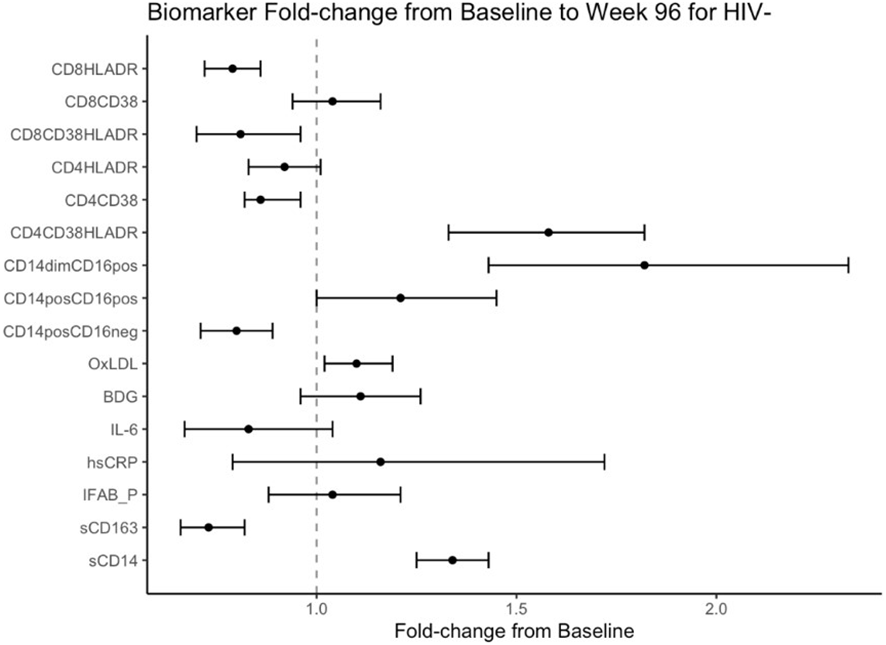
Fold Change from baseline over 96 weeks in HIV- participants. Point estimates and error bars reflect mean and 95% confidence intervals.

Mean fold change in biomarkers from baseline over time by study group are presented in **Table 2**; **Figures 1**, **2**. At week 96, hsCRP levels in PHIV decreased by 40% and remained stable in HIV-. At both time points, markers of systemic inflammation hsCRP and IL6 were not different between the arms (p≥0.19).

**Table 2:** Mean Fold change (97.5% Confidence Interval) from Baseline over 96 weeks by Group.

	PHIV			HIV-		
Biomarker	Mean Fold-Change (97.5% CI)	p-value	p-value (fdr)	Mean Fold-Change (97.5% CI)	p-value	p-value (fdr)
hsCRP (ng/mL)	0.60 (0.38, 0.94)	0.01	0.12	1.16 (0.79, 1.72)	0.39	0.54
IL6 (pg/mL)	0.85 (0.65, 1.10)	0.16	0.91	0.83 (0.67, 1.04)	0.07	0.33
OxLDL	1.01 (0.94, 1.10)	0.74	0.91	1.10 (1.02, 1.19)	**<0.01**	**0.04**
IFAB-P (pg/mL)	1.19 (1.03, 1.39)	0.01	0.08	1.04 (0.88, 1.21)	0.54	0.54
BDG (pg/mL)	1.38 (1.19, 1.57)	**<0.01**	**<0.01**	1.11 (0.96, 1.26)	0.08	0.33
sCD14 (pg/mL)	1.02 (0.91, 1.14)	0.60	0.91	1.34 (1.25, 1.43)	**<0.01**	**<0.01**
sCD163 (pg/mL)	0.69 (0.61, 0.79)	**<0.01**	**<0.01**	0.73 (0.66, 0.82)	**<0.01**	**<0.01**
%MNC: CD14^+^CD16^-^	0.95 (0.85, 1.06)	0.21	0.91	0.80 (0.71, 0.89)	**<0.01**	**<0.01**
%MNC: CD14^dim^CD16^+^	1.16 (0.87, 1.52)	0.28	0.91	1.82 (1.43, 2.33)	**<0.01**	**<0.01**
%MNC: CD14^+^CD16^+^	0.99 (0.79, 1.24)	0.91	0.91	1.21 (1.00, 1.45)	0.02	0.16
%CD4^+:^ CD38^+^	0.89 (0.83, 0.96)	**<0.01**	**<0.01**	0.86 (0.82, 0.96)	**<0.01**	**0.01**
%CD4+: HLA-DR^+^	0.77 (0.68, 0.86)	**<0.01**	**<0.01**	0.92 (0.83, 1.01)	0.04	0.23
%CD4^+:^ CD38^+^HLA-DR^+^	1.78 (1.49, 2.19)	**<0.01**	**<0.01**	1.58 (1.33, 1.82)	**<0.01**	**<0.01**
%CD8+: CD38^+^	0.96 (0.85, 1.08)	0.39	0.91	1.04 (0.94, 1.16)	0.38	0.54
%CD8^+:^ HLA-DR^+^	0.68 (0.62, 0.76)	**<0.01**	**<0.01**	0.79 (0.72, 0.86)	**<0.01**	**<0.01**
%CD8^+:^ CD38^+^HLA-DR^+^	0.88 (0.73, 1.05)	0.12	0.91	0.81 (0.70, 0.96	**0.01**	**0.04**

BDG, Beta D glucan; HLADR, Human leukocyte antigen DR; HS, high sensitivity; IFABP, intestinal fatty acid binding protein; IL, interleukin; MNC, monocytes.Bold values represent p values <0.05.

In contrast and despite the decreases in systemic inflammation in PHIV, I-FABP and BDG increased at week 96 and were 19% and 38% higher than baseline levels respectively; levels did not change significantly in HIV-. Formal comparisons of changes in I-FABP did not hold after FDR adjustment, however the consistency in magnitude and direction is suggestive of increased gut integrity damage over time in PHIV.

Oxidized LDL was significantly worse in HIV- at both times points (p ≤ 0.01) compared to PHIV and increased 10% in HIV- over 96 weeks but remained stable in PHIV.

### Changes in markers of monocytes and T-cell activation

At baseline, PHIVs had higher monocyte activation; higher sCD14 (p<0.01) and a higher proportion of non-classical CD14dimCD16+ monocytes (p<0.02) compared to these indices in HIV-. At week 96, sCD14 and proportion of all monocytes remained relatively stable in PHIV, however sCD14 and the proportion of CD14dimCD16+ monocytes increased by 34% and 80% respectively in HIV-.

sCD163 was significantly higher in HIV- at baseline and week 96 (p ≤ 0.01) and decreased in both arms at week 96, by 69% and 73% in PHIV and HIV- respectively.

At both time points, PHIVs had higher T cell activation (p ≤ 0.03: CD4+ and CD8+ T cells expressing HLA-DR) compared to HIV- children. Over 96 weeks, CD4+ T cell expressing HLA-DR and CD38 worsened in both groups by 78% in PHIV and 58% in HIV- (p<0.01 for both). CD8+T cell expressing both HLA-DR and CD38 decreased by 19% in HIV- (p=0.01).

### Role of sex and sexual maturity

Biomarkers were not different by sex among all participants at baseline or at week 96 (p≥0.09). Among PHIV participants, only hsCRP was higher in males at baseline (p=0.003), but all other biomarkers were similar between sexes at baseline and week 96 in PHIV and HIV- participants (p≥0.09).

Among all participants, higher sexual maturity rating or tanner stage correlated with higher hsCRP (r=0.16, p=0.05) and lower fungal translocation (r=-0.17, p=0.02).

### Correlations

At baseline, markers of systemic inflammation (hsCRP), and fungal translocation (BDG), correlated with monocyte and T- cell activation in PHIV (r=0.24 - 0.33, p<0.03). In HIV-, however, only markers of systemic inflammation (hsCRP and IL6) positively correlated with CD8 activated T cells (r= 0.24-0.28; p<0.03). Only in PHIV, at both time points, oxidized LDL was inversely associated with monocyte and activated T cells (r ranges between -0.44 and -0.15, p<0.01).

### Role of ART and HIV factors

At baseline, there was no correlation between ART duration, PI or NNRTI use and plasma, cellular markers of monocyte activation or T-cell activation (p≥0.32). Lower nadir CD4+ cell count correlated with proportions of traditional (CD14+CD16-) and non-classical monocytes (r=0.27-0.28), as well as CD4 and CD8+ T-cells expressing HLA-DR and CD38 (r= 0.24-0.28; p ≤ 0.03 for all).

We examined the effects of switching ART regimen during the study period, in separate models for each marker. The only significant interaction between medication switch and study duration was for sCD163. The results of a combination of coefficients, medication switch plus interaction term, showed that there was a significant increase at week 96 for participants who switched ART during the study (β=0.29, 95% CI: 0.07, 0.50, p= 0.01);. At week 96, sCD163 was lower for those who continued the same medication and those who switched medications had higher sCD163.

We then looked specifically at the effects of switching to dolutegravir (**Table 3**) Switching to dolutegravir at week 96 was significantly associated elevated levels of sCD163 (β=0.4, 95% CI:0.14,0.57, p<0.01) and oxidized LDL (β=0.08, 95%CI: 0.009, 0.16, p=0.03) and had a marginal effect in reducing sCD14 levels and IL6.

**Table 3:** Analysis of association between dolutegravir switch during study and changes in inflammatory biomarkers.

Biomarker	Regression coefficient (95% CI)	p-value
hsCRP (ng/mL)	0.11 (-0.14, 0.37)	0.39
IL6 (pg/mL)	0.39 (-0.02, 0.81)	0.06
OxLDL	0.08 (0.009, 0.16)	**0.03**
IFAB-P (pg/mL)	0.15 (-0.08, 0.39)	0.20
BDG (pg/mL)	-0.004 (-0.017, 0.007)	0.43
sCD14 (pg/mL)	-0.12 (-0.26, 0.011)	0.07
sCD163 (pg/mL)	0.40 (0.211, 0.59)	**<0.01**
%MNC: CD14^+^CD16^-^	-0.05 (-0.14, 0.05)	0.33
%MNC: CD14^dim^CD16^+^	-0.007 (-0.06, 0.04)	0.77
%MNC: CD14^+^CD16^+^	0.05 (-0.18, 0.12)	0.14
%CD4^+:^ CD38^+^	1.06 (-0.388, 2.52)	0.15
%CD4+: HLA-DR^+^	1.32 (-0.53, 3.18)	0.16
%CD4^+:^ CD38^+^HLA-DR^+^	2.3 (-1.3, 5.9)	0.21
%CD8+: CD38^+^	0.32 (-1.66, 2.32)	0.75
%CD8^+:^ HLA-DR^+^	0.34 (-0.98, 1.66)	0.61
%CD8^+:^ CD38^+^HLA-DR^+^	4.22 (-0.77, 9.23)	0.09

GEE models ran separately for each biomarker and included terms for observation time, switch to dolutegravir and an interaction terms dolutegravir switch x time. The interaction term coefficient, 95% CI and p values are the values presented here. BDG, Beta D glucan; HLADR, Human leukocyte antigen DR; HS, high sensitivity; IFABP, intestinal fatty acid binding protein; IL, interleukin; MNC, monocytes. Bold values represent p values <0.05.

## Discussion

Our data highlight that children with PHIV display ongoing CD4 T cell immune activation and evidence of ongoing gut structural damage despite long term viral suppression on ART. Although systemic inflammation improved in PHIV, levels of I-FABP, a marker of enterocyte inflammation, and BDG, a marker of fungal translocation, increased over time.

PHIV is lifelong and started *in utero*, at birth or shortly thereafter. People with PHIV have endured HIV and ART throughout their immunological development. Non-perinatal HIV in adults is typically acquired after immunological development is complete. Many children and youth with PHIV started ART with more toxic combination regimens (e.g., thymidine analogues), whereas more recently infected adults or adolescents have access to less toxic ART (e.g., integrase strand transfer inhibitors). Repeat challenges to the developing immune system, including HIV and ART, may lead to an immune disturbances and trajectory not seen in adults with HIV. There is a dearth of research assessing immune dysfunction in perinatal HIV on ART as they age towards adulthood.

Similarly to adults with HIV, we found that some indices of immune activation and systemic inflammation improved over time in PHIV with ART (11), yet, levels may not reach those of populations without HIV (12, 13). To our knowledge, this is the first comprehensive and longitudinal study investigating changes in inflammatory biomarkers and T-cells and monocyte activation in PHIV on ART in sub-Saharan Africa and in age and sex matched children without HIV.

Most participants in our cohort initiated ART early, after 2 years of age, however, this is likely too late to eliminate residual inflammation during ART. Evidence from the RV254/RV304 in a cohort of adults with HIV suggest that ART initiation during HIV seroconversion may mitigate or eliminate persistent inflammation (14). Findings in adults with HIV suggest that ongoing viral replication is likely not responsible for elevated levels of inflammation and immune activation once plasma viremia is consistently suppressed by ART and the strongest associations found for markers of HIV persistence on ART were pre-ART levels of plasma, and cell associated HIV-1 DNA (15). These findings suggest that strategies to reduce inflammation and immune activation in HIV need to focus on 1) reversing the damage induced by HIV prior to ART initiation and/or 2) on the timing between seroconversion and ART initiation. Perinatal HIV infection presents a unique opportunity to decrease the reservoir and limit ongoing inflammation through early ART (16) and studies within the International Maternal, Pediatric, Adolescent AIDS Clinical Trials (IMPAACT) network are currently examining the long-term clinical, immunologic and virologic profiles of children who received early combination ART (ART initiated within 12 weeks of birth). In a cohort of 440 infants who initiated pre-emptive ART within 48 hours, 34 of whom were diagnosed with *in utero* HIV infection, and continued ART with virologic suppression for 2 years, the authors found that 89% tested HIV-1 antibody negative and 70% had non-detectable cell-associated HIV-1 DNA through age 2 years (17). These findings suggest that infants with very early ART initiation may achieve restricted HIV-1 reservoirs.

Persistent immune activation plays a key role in HIV pathogenesis and co-morbidities (18–20) and may be a result of ongoing inflammation triggered by gut dysfunction and microbial translocation (21). Our findings support this hypothesis as well as earlier observations made by our group in adults living with HIV that 1) intestinal integrity markers like I-FABP do not improve overtime despite successful ART and that 2) fungal translocation as measured by BDG is associated with inflammation and immune activation (22). BDG is known to be highly immunogenic (23, 24). Similar to adults with HIV, we demonstrated a correlation between serum levels of BDG and markers of monocyte and T cell activation that was not seen in uninfected controls. This suggests that fungal translocation from an inflamed gut mucosa may contribute to the ongoing inflammation and immune activation seen in PHIV despite viral suppression on ART.

We have previously found that oxLDL is a main driver of systemic inflammation and monocyte activation in adults (9, 25), therefore we assessed the correlation between oxLDL and measured biomarkers. We found that lower oxidized LDL was associated with a weak but consistent increase in T cell and monocyte activation throughout the study. Oxidized LDL is an oxidized form of LDL cholesterol that may cause endothelial and smooth muscle cell dysfunction, can modulate innate and adaptive immunity or be taken up by macrophages to form atherosclerotic plaques (26). OxLDL is associated with coronary heart disease in adults (27, 28). In the Randomized Trial to Prevent Vascular Events in HIV (REPRIEVE), the largest primary CVD prevention trial in HIV, higher levels of oxLDL was associated with male sex, residence in high-income regions, white race and higher BMI (29). These findings highlight how there might be differences in systemic immune pathways in the global North and South. However, our findings are contrary to what has been found in adults living with HIV. We hypothesize that this may be due to the fact that Ugandan PHIV in our study had normal BMI and lower LDL cholesterol (5), which may explain why oxidized LDL may not appear to be in the pathway of immune activation in younger populations.

Among the uninfected participants, we found that sCD163 levels were higher than levels in PHIV and that significant markers of oxidative stress (oxidized LDL), monocyte activation (sCD14), and the proportions of patrolling monocytes and activated CD4+ T cells all increased over 96 weeks. Our findings highlight non-HIV sources of inflammation that are likely endemic to Sub-Saharan Africa. Exposure to multiple co-occurring adversities likely influence inflammation in this setting and may include exposure to social adversity, such as economic hardship, food insecurity, chronic stress (30–34) and exposure to environmental adversity, such as ambient air pollution – which has been increasing in SSA over the past decade (29) – has also been linked to inflammation (35). Although acute co-infections were exclusionary in this study, both groups in this study were exposed to high levels of economic insecurity, and PHIV were more likely to lack access to clean water and electricity. Several factors may limit the natural progression of inflammation in PHIV and buffer the adverse effects of socioeconomic adversity in addition to early and successful ART, and may include access to routine health care and the anti-inflammatory properties of co-trimoxazole (36).

There are limited data on assessing immune dysfunction in PHIV on ART as children age and go through puberty. In this study, 30% of participants underwent puberty during the study period. This is relevant as the function of the immune system peaks during puberty (37). We have previously reported a lack of sex related differences in immune markers in a younger Ugandan cohort (38) (Kamari paper) and hypothesized that our findings were likely secondary to the prepubertal stage of the participants. Similarly, in this study, we found a lack of sex differences both at baseline and week 96, regardless of HIV status. However, women living with HIV have been reported to have higher systemic immune activation and decreased gut integrity markers compared to men (39–43). We hypothesize that our findings could be secondary to a combination of factors including differences in microbiomes, early acquisition of HIV during immune development may lead to similar immune setpoints regardless of sex differences, and or sex-specific genetic and epigenetic regulation may not become relevant until after puberty and could explain the relationship between tanner staging and higher hsCRP.

Our study is the first to look at the effect of switching to dolutegravir on markers of inflammation in PHIV youth in this setting. Participants in our study were switched to 3TC, TDF and DTG during the study period following regimen optimization based on the Ugandan Ministry of Health HIV Guidelines (10). We found that most markers did not change when switching to DTG and that only sCD14 and IL6 slightly improved while sCD163 and oxidized LDL worsened. Interpretation of our results must be done with caution as our analysis was done in a non-randomized population with different ART backbones. These findings, however, are consistent with data from adult studies in virally suppressed participants on ART switching to a DTG-based regimen. In a randomized open-label Phase IIIb study in adults virally suppressed on ART randomized to switch to ABC, DTG and 3TC or continue on their current ART regimen (either 2 NRTIs plus either a PI or an NNRTI), participants who switched to a DTG based regimen had significant decrease in sCD14 and I-FABP and a small but not significant increase in sCD163 (44). In a retrospective case-cross over study where virologically suppressed patients were switched from 3TC and a PI to 3TC and DTG, similarly to our findings, sCD14 decreased, while other markers remained stable (45). Further studies are needed to assess whether DTG will have a differential impact on inflammatory biomarkers or any evidence on downstream clinical outcomes in this young population especially as DTG is now used as the preferred ART for children and adolescents worldwide.

Our study includes well-characterized cohorts of adolescents with and without HIV that are age and gender matched and are from the same country and urban area for comparison. In addition, our study is strengthened by the longitudinal design and the long-standing viral suppression in PHIV. The biomarkers selected for this analysis are likely part of an interrelated group and may consist of multiple steps and feedback loops, we therefore corrected for multiple comparisons. There are a few limitations to our study including the lack of assessment of the composition of the gastrointestinal microbiome and mycobiome, or of bacterial translocation. Although we excluded participants with active infections, we did not assess for potential helminthiasis or perform nutritional assessments, all of which could affect the microbiome. In addition, a little over half of the participants had an ART regimen switch during the study period.

In conclusion, our study showed that children who acquired HIV perinatally have evidence of ongoing T cell activation, damage to the gut integrity and fungal translocation despite ART and viral suppression over 2 years. Further research is warranted in this population to investigate the links between intestinal barrier function, intestinal microbiota composition, and immune activation, and the implications of life long exposure to the consequences of decreased gut-barrier function on potential comorbidities in HIV-infected children. In addition, our findings highlight the need for continued research on newer ART regimens and early ART initiation in PHIV to decrease inflammation and prevent long-term comorbidities.

## Data availability statement

The raw data supporting the conclusions of this article will be made available by the authors, on a case by case basis.

## Ethics statement

The studies involving human participants were reviewed and approved by University Hospitals Medical Center IRB and Joint Clinical Research Center IRB. Written informed consent to participate in this study was provided by the participants’ legal guardian/next of kin.

## Author contributions

SD-F and GM conceptualized and designed the study. Data collection was performed by CG, RN, and SD-F. Data analysis and interpretation was performed by AS, MS and SD-F. Immune assays were performed by KA and NF. The article was drafted by SD-F and revisions and final approval by all co-authors. All authors contributed to the article and approved the submitted version.
